# Plant traits linked to field-scale flammability metrics in prescribed burns in *Eucalyptus* forest

**DOI:** 10.1371/journal.pone.0221403

**Published:** 2019-08-26

**Authors:** Bianca J. Tumino, Thomas J. Duff, Jason Q. D. Goodger, Jane G. Cawson

**Affiliations:** 1 School of Ecosystem and Forest Sciences, The University of Melbourne, Burnley, Victoria, Australia; 2 School of BioSciences, The University of Melbourne, Parkville, Victoria, Australia; University of the Balearic Islands, SPAIN

## Abstract

Vegetation is a key determinant of wildfire behaviour at field scales as it functions as fuel. Past studies in the laboratory show that plant flammability, the ability of plants to ignite and maintain combustion, is a function of their traits. However, the way the traits of individual plants combine in a vegetation community to affect field flammability has received little attention. This study aims to bridge the gap between the laboratory and field by linking plant traits to metrics of field-scale flammability. Across three prescribed burns, in *Eucalyptus*-dominated damp and dry forest, we measured pre-burn plant species abundance and post-burn field flammability metrics (percentage area burnt, char and scorch height). For understory species with dominant cover-abundance, we measured nine traits that had been demonstrated to influence flammability in the laboratory. We used fourth-corner ordination to evaluate covariation between the plant traits, species abundance and flammability. We found that several traits covaried at the species level. In some instances, these traits (e.g. specific leaf area and bulk density) could have cumulative effects on the flammability of a species while in other instances (e.g. moisture and specific leaf area) they may have counteractive effects, assuming trait effects on flammability are akin to previous research. At field scales, species with similar traits tended to co-occur, suggesting that the effects of individual traits accumulate within a plant community. Fourth-corner analyses found the trait-field flammability relationship to be statistically significant. Traits significantly associated with increasing field flammability metrics were: bulk density (negatively associated) and hydrocarbon quantity, specific leaf area and surface area to volume ratio (all positively associated). Our study demonstrates that some traits known to influence flammability in the laboratory can be associated with field-scale flammability metrics. Further research is needed to isolate the contributions of individual traits to understand how species composition drives forest flammability.

## 1. Introduction

Vegetation acts as fuel in a wildfire and thus a plant’s ability to ignite and maintain combustion (plant flammability; [1]) is likely to be a key determinant of how plant communities burn at field scales. Vegetation communities dominated by plants that are of high flammability may burn faster or more intensely under similar weather and terrain conditions [2,3]. Conversely, if the dominant species are of low flammability then wildfires may be less intense, slower moving or not burn at all [4,5]. Understanding how the characteristics of vegetation influence field-scale flammability (the ability of vegetation communities to ignite and maintain combustion) may be important not only for predicting individual wildfire behaviour, but for predicting how changes to species composition may influence the flammability of the forest and therefore fire regimes [6–8].

An array of traits of living plants have been shown to influence the flammability of individual plants or plant parts in laboratory settings (Table 1). Traits shown to negatively influence plant flammability include fuel moisture content [9,10], ash content [11,12] and leaf thickness [13]. Conversely, traits shown to positively influence flammability include volatile oil content [14], specific leaf area [15], leaf surface area [16] and surface area to volume ratio [17]. The effects of the bulk density of live material on flammability has not been determined in the laboratory. Fuel moisture has been the most widely studied of the traits and dead fine fuel moisture is a key consideration when predicting forest fire danger [18,19]. The remaining live plant traits described in Table 1 are not a core consideration of operational fire behaviour modelling systems (one exception being the inclusion of live woody and herbaceous surface area to volume ratio in Rothermel’s [20] surface fire spread model). Instead, a number of empirical fire spread models specific to particular vegetation-types have been developed in Australia (for example, the buttongrass model [21], the shrubland model [22] and the dry eucalypt model [23]), which likely integrate the effects of plants and species composition on fire behaviour. The limited consideration of plant traits in models likely reflects the challenge of quantifying links between plant traits and fire behaviour at field scales and thus limiting the understanding about how the traits of individual species interact and combine to influence the flammability of whole vegetation communities [24–26]. A single trait on an individual plant is unlikely to drive fire behaviour at field scales but when the traits of many plants combine, or a single species is dominant, the magnitude of effect may be substantial (e.g. [2]).

**Table 1:** Summary of plant flammability findings for laboratory studies of live plant traits.

Plant trait	Definition of trait	Correlation with plant flammability	Works describing the plant trait and plant flammability association
Ash quantity (%)	Percent of mineral content remaining after combustion determined as a ratio of oven dry weight	(-) combustibility	[11,12,27–30]
Bulk density^1^ (g cm^3^)	Amount of oven dry biomass per volume of airspace	Undetermined in the laboratory	[24,25,31,32]
Extractives^2^ (mg gDW^1^)	Amount of volatile compounds including waxes, fats, oils and terpenes. Typically, only terpenes are considered, or no distinction is made between terpenes and other volatile compounds.	(+) ignitability (+) combustibility	[12,14,33–37]
Fuel moisture content^3^ (%)	Percent of water in a plant organ determined as a ratio of dry weight.	(-) ignitability (-) combustibility	[16,17,30,37–41]
Specific leaf area (cm^2^ g^-1^)	The one-sided area of a fresh leaf, divided by its oven dry weight	(+) ignitability	[15,16,42]
Surface area (cm^2^)	The total area of a plant organ, typically leaves	(+) ignitability	[16,43–45]
Surface area to volume ratio (cm^-1^)	Ratio between surface area and total volume of a plant organ	(+) ignitability (+) combustibility	[17,29,46,47]
Thickness (cm)	Width of the plant organ	(-) ignitability	[13,48]

^1^ Bulk density has a parabolic relationship with flame spread rate; it can be aeration or fuel limiting and its effect depends on the fuel layer being investigated. It has been extensively studied in the laboratory for leaf litter.

^2^ Several plant flammability studies that include volatile compounds are inconclusive about their importance.

^3^The effects of fuel moisture content (FMC) is not always consistent, as some live fuels can burn intensely at high FMC.

The direction of correlation with the plant trait and the flammability metric tested is shown in brackets

Most research on plant flammability has been done in the laboratory, focusing on the relationship between plant traits and the combustion of individual leaves, plant parts or reconstructed fuel beds. Such studies have been designed to: identify which traits are most important to a particular combustion characteristic [37,49,50]; use traits to score and compare the flammability of different plant species [47,51] and vegetation types [12,36]; or use traits to explain changes in landscape flammability as a result of changes in community species composition [52]. Practicalities of working within a laboratory mean the scale of research is often restricted to plant parts rather than whole plants or groups of plants and the heat source applied to the vegetation may not be analogous to wildfire conditions [53]. Additionally, most studies only consider the effects of a small number of traits [14,54], making it difficult to determine the combined effect of multiple traits. As such, laboratory-based plant flammability research has been criticised for having limited applicability to field-scale flammability [31]. Recent studies have involved cross-scale comparisons between the flammability of individual leaves and litter beds [50,55] but the challenge of translating the results of laboratory studies to landscape scales remains [56,57]. Given the potential magnitude of the effect of species on field-scale flammability, being able to understand how plant-level traits link to field-scale fire behaviour is critical to understanding fire at larger scales.

In response, our study sought to bridge the gap between the laboratory and field by linking plant traits of dominant understory species to field-scale flammability metrics in three prescribed burns in damp and dry *Eucalyptus* forests of south-eastern Australia. These forests consist of a *Eucalyptus* tree overstory and an understory dominated by shrubs and ferns [58] (Table 2). Specifically, we asked:

1. Do plant traits co-vary within a species?
2. Do co-occurring plant species exhibit similar plant traits?
3. Is there a relationship between plant traits and flammability metrics at field scales?

**Table 2:** List of dominant plant species measured in this study, and the percentage of plots in which the species are present in the Damp (n = 110), Shrubby Foothill (n = 67) and Heathy Dry (n = 10) forest types.

Species name	Form	Damp (%)	Shrubby Foothill (%)	Heathy Dry (%)	Max plot cover (%)
*Acacia mucronata*	Shrub	4	19	0	20
*Acacia verticillata*	Shrub or small tree	25	7	0	80
*Bedfordia arborescens*	Small open tree or large shrub	23	22	0	50
*Calochlaena dubia*	Fern	15	12	0	90
*Coprosma quadrifida*	Shrub	57	12	70	50
*Correa lawrenciana*	Large shrub	3	0	0	65
*Cyathea australis*	Fern	24	7	30	80
*Goodenia ovata*	Shrub	25	7	40	85
*Hakea decurrens*	Shrub	0	7	0	40
*Kunzea ericoides*	Shrub	3	1	10	30
*Lepidosperma elatius*	Sedge	20	9	20	80
*Monotoca scoparia*	Shrub	3	12	20	40
*Olearia argophylla*	Large shrub or small tree	15	0	10	75
*Olearia lirata*	Shrub	19	6	0	65
*Pimelea axiflora*	Shrub	12	9	10	15
*Platylobium formosum*	Shrub	10	24	0	40
*Polystichum proliferum*	Fern	5	0	0	40
*Pomaderris aspera*	Erect shrub or small tree	28	15	0	75
*Pteridium esculentum*	Fern	71	78	50	70
*Pultenaea juniperina*	Shrub	11	51	10	60
*Pultenaea muelleri*	Shrub	9	21	0	75
*Spyridium parvifolium*	Shrub	13	6	0	45
*Tetrarrhena juncea*	Grass	73	84	70	80

Figures are based on data collected prior to the burns.

## 2. Methods

We used a multi-scale assessment to link plant traits to community-level flammability. Within three prescribed burns we undertook vegetation surveys pre-burn, measured burn outcomes (hereafter referred to as flammability metrics) as an indicator of field-scale flammability, and measured plant traits in the laboratory for the dominant understory species across the burns. We analysed the data using RLQ and fourth-corner analysis; these approaches provide for matrices of plant traits, plant community composition and environmental properties to be combined to determine trait/environment relationships [59].

### 2.1 Site description

**Fig 1:**
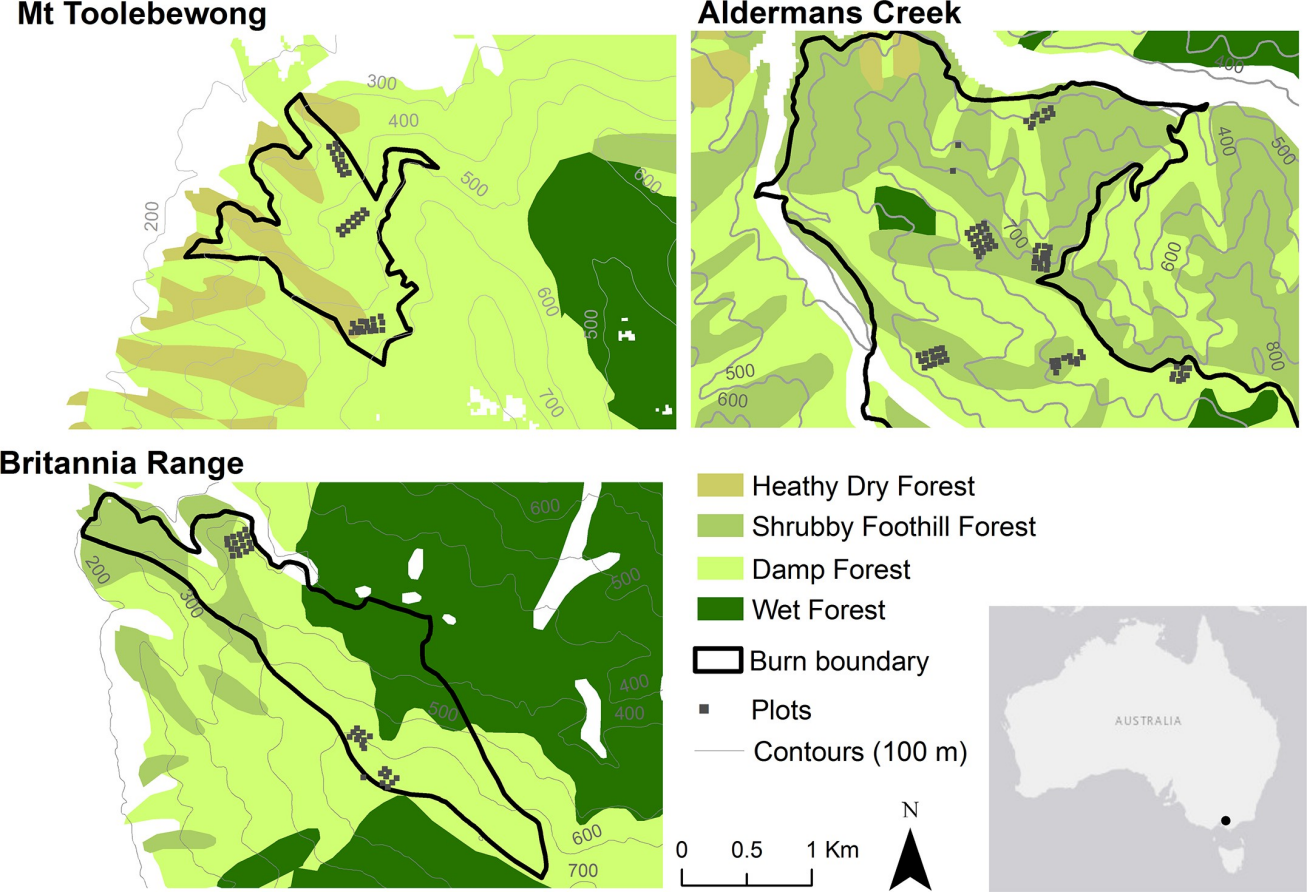
Plot layout within the three prescribed burns (Mt Toolebewong (37° 42' S, 145° 33' E); Alderman’s Creek (37° 44' S, 145° 58' E); and Britannia Range (37° 46' S, 145° 40' E)) in Victoria, Australia.

We undertook the study in three prescribed burns in *Eucalyptus* forests in the central highland region of Victoria, Australia (Fig 1). The burns were located in Yarra Ranges National Park (Aldermans Creek: 37° 44' S, 145° 58' E; burn size 2024 ha), Mt Toolewbong State Forest (Mt Toolebeong: 37° 42' S, 145° 33' E; burn size 149 ha) and Yarra State Forest (Britannia Range: 37° 46' S, 145° 40' E; burn size 268 ha), and were conducted by the Department of Environment, Land, Water and Planning (DELWP) as part of their fuel management program to reduce wildfire risk. Burnt area coverage is not intended to be complete (coverage within a completed burn has been found to average ~75%; [60]), with unburnt areas occurring naturally as a function of environmental conditions and vegetation properties. Research was conducted under permit number 10007375, issued jointly by Parks Victoria and DELWP.

**Fig 2:**
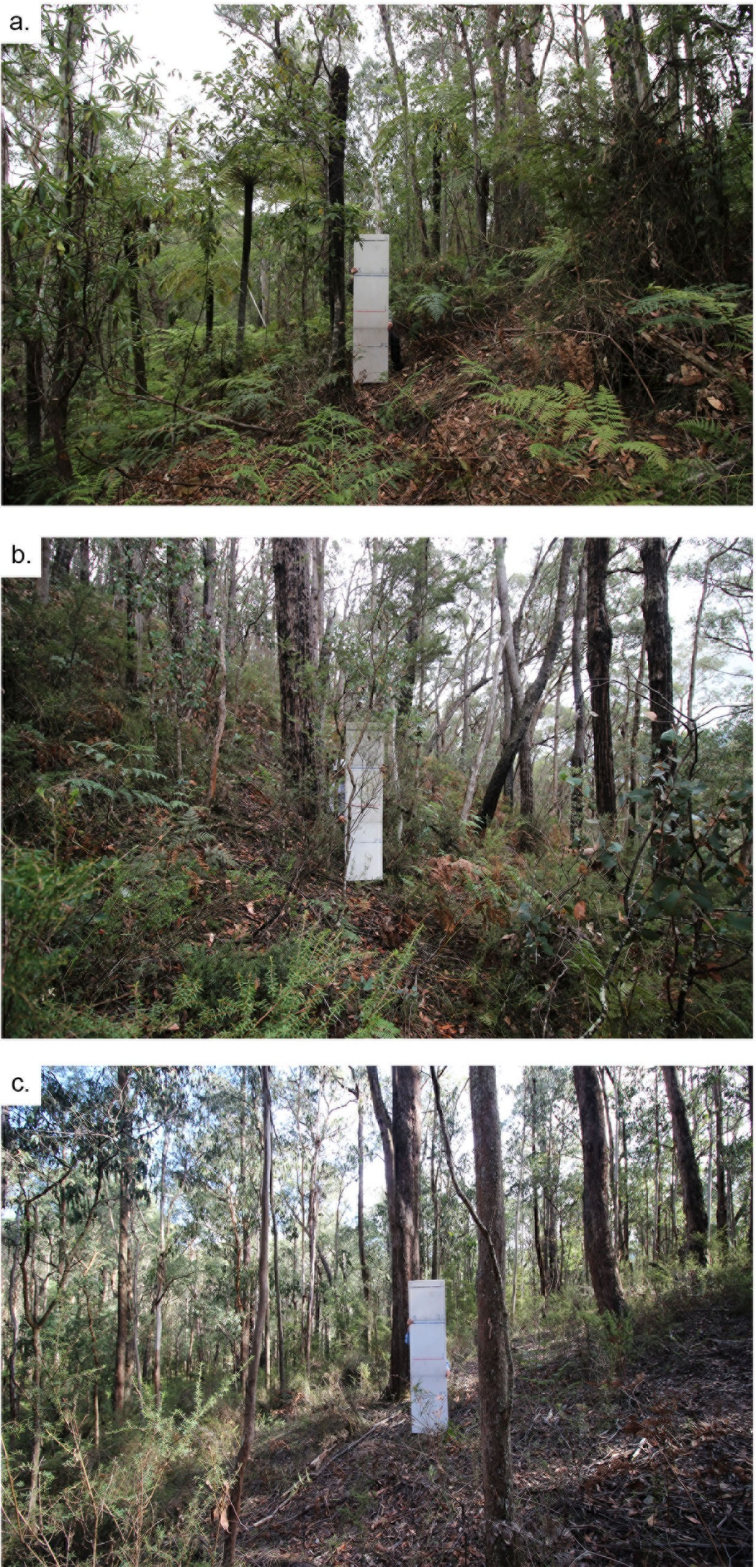
Photos illustrating the typical appearances of the understorey vegetation in (a) Damp forest, (b) Shrubby foothill forest, and (c) Heathy Dry Forest.

The climate of the region is temperate, without a dry season and with a warm summer, according to the Köppen-Geiger climate classification [61]; the mean annual rainfall across the study sites is 734 mm [62]. Terrain is rugged with strong effects of aspect and hillslope position on vegetation structure and moisture availability [63,64]. There were three dominant vegetation types within the burns [65]: Damp Forest, Shrubby Foothill Forest and Heathy Dry Forest (Fig 2). Although the vegetation types share many plant species in common, there are substantial differences in their frequency of occurrence (Table 2). Wildfires are common, with tolerable fire intervals of 25–150 years for Damp Forest, 25–100 years for Shrubby Foothill Forest and 15–45 years for Heathy Dry Forest [66].

Burning occurred in autumn (March to May) of 2016 and 2017 under mild weather conditions (S1 Table). During the burns, maximum daytime temperatures ranged from 19 to 27 ^o^ C, minimum relative humidity ranged from 23 to 55%, maximum Forest Fire Danger Index (FFDI) [67] ranged from 6 to 22, maximum Keetch-Byram Drought Index (KBDI) [68] ranged from 101 to 140. Burns were conducted by local land management agencies and ignited with hand-held drip torches from external edges and aerial incendiaries for internal ridgelines. All ignition attempts were lit as backing fires; burning downhill and with minimal wind effect across sites.

### 2.2 Field sampling

Prior to burning we surveyed vegetation in a total of 199 plots across the three prescribed burns. Plots were 5 m in radius and spaced at least 50 m apart and placed from ridgeline to midslope to capture environmental gradients caused by aspect and hillslope position. Within the plots we visually assessed the percent cover of understorey species with 5% or more cover. We assessed understorey vegetation, not overstory trees, because typically only the understorey is burnt under prescribed burning conditions.

Following the burn, we assessed plot-level flammability by visually estimating the percent of surface area burnt within the plot, the maximum char height and maximum scorch height. We defined char height as the maximum height of blackened leaves or charred non-fibrous bark and scorch height as the maximum height of dead (browned) leaves on trees and shrubs. Char and scorch heights provide an indication of flame height [69] and hence the combustibility of the vegetation within the plot [7,53,70]. The proportion of surface area burnt provides a measure of the ignitability of the vegetation within the plot [71]. Not all plots had exposure to ignitions during the burn operations—of the 199 plots evaluated, 124 were exposed to ignitions and remained suitable for analysis (S1 Dataset).

### 2.3 Species trait measurements

We assumed that the most abundant species would have a dominant influence on the flammability of the vegetation within a plot. As such, we undertook trait analysis for plant species that were most common; occurring in a minimum of 10 plots or had a cover abundance exceeding 40% in at least one plot. The one exception was *K*. *ericoides* which was found in 5 plots with a maximum cover of 30%—it was chosen as a species of interest as it rapidly colonizes disturbed areas and is actively invading native vegetation in south eastern Australia [72,73]. In total there were twenty-three species selected for trait analysis out of a total of 108 species (Table 2). Of the 124 plots exposed to ignitions during burn operations, 117 of these had our combination of species as the plot’s dominant flora and were suitable for final analysis (S1 Dataset).

We collected samples of the dominant plant species from in, and adjacent to, the Britannia Range burn in April 2017. For each species, we collected samples from five individuals plants (as per [42]). Plant material was collected by working from the leaf or leaf-like structure, to the point where it would no longer be considered as live fine fuel i.e.Less than 2 mm diameter [69,74]. We considered leaflets as the measurement unit for species with compound leaves (e.g. ferns). We sampled bulk density by removing fine fuels within a 20 cm^3^ cube placed within the plant canopy midway between the base and top of the canopy for that plant.

We removed any water present on the plant surface from dew or rain using paper towel before sealing the specimen in plastic zip lock bag and storing it in a cool box for transport. Within a few hours of sample collection, all samples were refrigerated at 4°C until measurements were made, with the exception of the samples collected for the extractive analysis, which were stored at -80° C. We completed moisture sensitive measurements within 24 h of field collection. Table 3 outlines the methods applied for flammability trait quantification. Measurements were conducted on all parts of the fine fuel sampled for each the species, with the exception of surface area (SA) and extractives. We measured SA on individual leaves or leaflets to make the data comparable with measurements in other studies and we measured extractive content on leaves or leaflets as there are few extractives stored within other plant organs [14,75].

**Table 3:** Description of the plant trait measurement methods.

Plant trait	Measurement method	Studies with similar techniques
Ash quantity^1^ (%)	Sample was ground and 3 g was ‘ashed’ in a muffle furnace at 550 ºC for 30 minutes before the residue was weighed.	[11,12,27–29,76–78]
Bulk density (g cm^3^)	Samples collected in field from within a 20 cm^3^ cube were oven dried and weighed to calculate mass of dry fine fuel per unit volume of space.	[7,51]
Extractives: Terpenes and Hydrocarbons (mg/g DW)	Standard hexane extraction method. The term terpene is used as a collective definition for monoterpenes and sesquiterpenes, and the term hydrocarbon defines longer chain waxes and fats.	[79]
Fuel moisture content (%)	Fine fuels were dried in an oven at 105°C for 48 hours and weighed twice, 24 hours apart, to ensure they reached constant mass. Fuel moisture was calculated as the percent of water as a function of oven dry weight.	[39,80]
Specific leaf area (cm^2^ g^-1^)	Typically calculated as one-sided surface area of a fresh leaf divided by its oven dry weight. For the purposes of this study, we consider *total* surface area divided by oven dry weight. For surface area calculations see “surface area to volume ratio”.	[42,50]
Surface area (cm^2^)	Length and width were measured at the widest part of a flattened leaf or leaflet. One-sided surface area was determined by multiplying length and width.	[16,17,81]
Surface area to volume ratio (cm^-1^)	The LI-COR LI-300C area meter was used to measure one-sided leaf surface area (cm^2^). Material that was cylindrical was measured separately using the curved surface area equation of a cylinder. Volume (cm^3^) was calculated using water displacement.	[42,47,50,81]
Thickness (cm)	Electronic callipers were used to measure at the leaf’s widest part, and at a point two-thirds the distance from the edge to the mid-rib.	[16,17,42,81]

^1^ Silica-free ash has been recommended as a better comparison between species, however, total ash quantity is likely to be a sufficient measure for these plants because silica is minimal in non-grass species, and there is usually a strong correlation when comparing ash and silica-free ash values.

### 2.4 Data analysis

**Fig 3:**
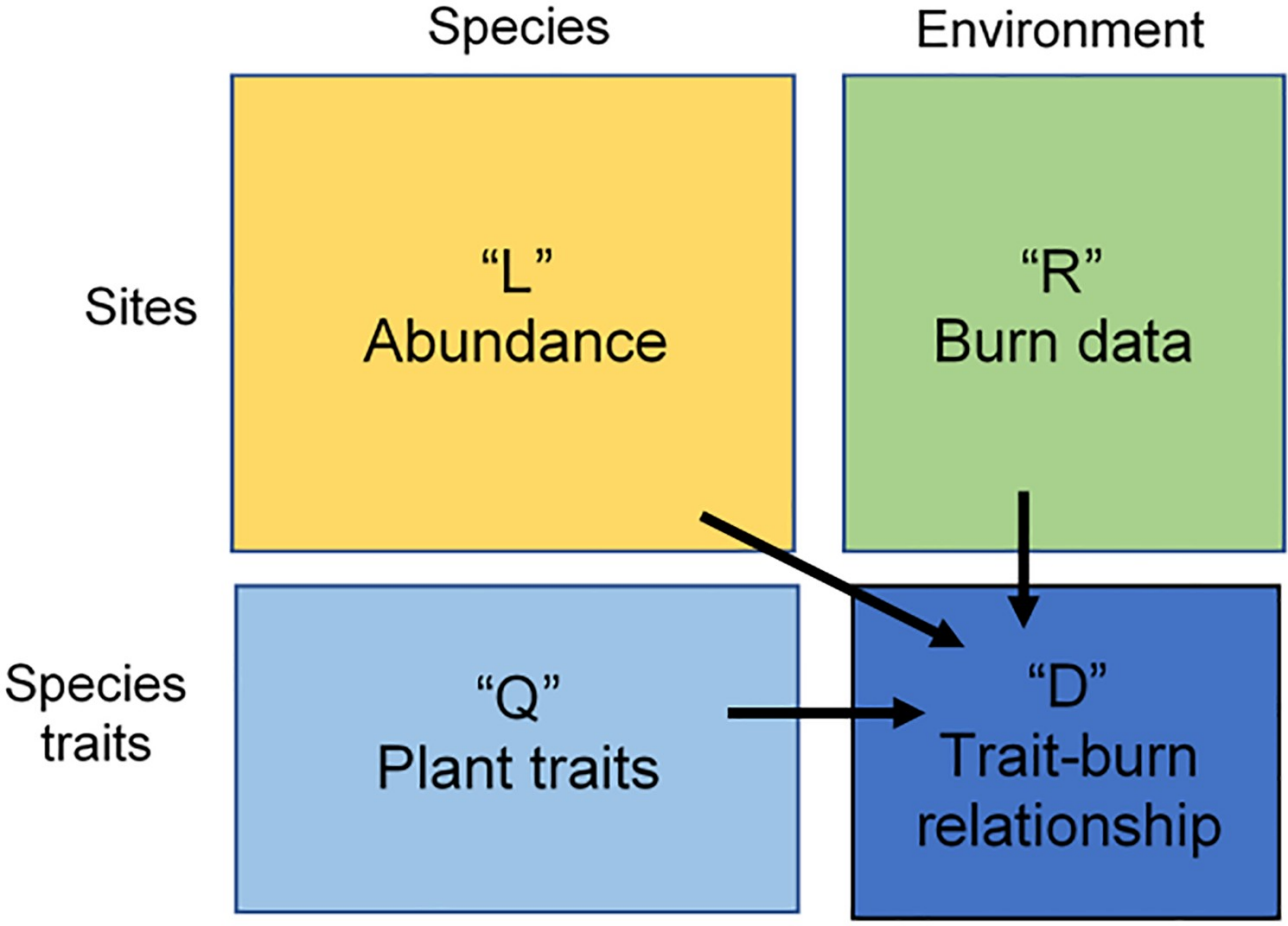
Graphical representation of the RLQ and fourth-corner analysis (adapted from [84] and [85]). The method combines abundance (L), trait (Q) and burn (R) data, to determine the trait and field flammability relationship (D).

To quantify associations between traits on the same species, we calculated the Pearson Correlation Coefficients between each pair of traits. As terpene content and SA data were highly skewed, they were normalised using log transformation prior to analysis. To evaluate the link between plant traits and flammability metrics, we used the complementary methods: RLQ and fourth-corner analyses [59]. These analyses are designed to link species trait data (Q) to environmental measurements (R) using species composition data (L). The methods are designed to determine the fourth-corner trait-environment relationship (D) by linking ordinations of site-environment (R), species-trait (L) and species-site (Q) using a constraining process (Fig 3). The environment data (R) is conventionally used to represent biophysical properties (soil, rainfall etc.). However, we used it to represent the site measured flammability metrics. A preliminary step in the RLQ is the separate analysis of each table of data using principal component analysis (PCA) for the flammability metrics (R) and trait (Q) tables and correspondence analysis for the species composition (L) table. RLQ is an analysis of the L table constrained by R and Q to provide for the creation of the trait-burn table, D. In contrast, the fourth-corner approach measures and tests the multiple associations between one trait and one environmental variable. A multivariate test brings the separate analyses together to evaluate the global significance of the trait and field flammability relationship; in this test we used 49,999 permutations. We undertook all statistical analyses and graphical outputs using R (version 3.4.1; [82]) and the ade4 package [83] for the RLQ and fourth-corner analysis.

## 3. Results

There was a strong positive correlation between fuel moisture content (FMC) and specific leaf area (SLA) (r = 0.82; Table 4), and a moderate positive correlation FMC and SA (r = 0.50; Table 4), suggesting that species with larger leaves (per unit mass of fuel) tended to have higher moisture contents. FMC was also strongly positively correlated with hydrocarbon content (r = 0.62; Table 4). High negative correlations were observed between bulk density and SA, SLA and surface area to volume ratio (SVR) (r = -0.44, r = -0.56 and r = -0.48 respectively; Table 4), suggesting species with larger leaves had less mass of dry fine fuel per unit volume of space. Thickness and ash quantity were not strongly correlated with any other trait.

**Table 4:** Pearson’s correlation coefficients between plant traits for the 23 most dominant plant species within the prescribed burns.

	Ash quantity	Bulk density	Hydro-carbon	Fuel moisture content	Surface area	Specific leaf area	Surface area to volume ratio	Terpene
Bulk density	0.12							
Hydrocarbon	-0.08	-0.29						
Fuel moisture content	0.18	-0.41	0.62**					
Surface area	0.39	-0.44*	0.43*	0.50*				
Specific leaf area	0.16	-0.56**	0.50*	0.82***	0.56**			
Surface area to volume ratio	0.12	-0.48*	0.12	0.26	0.28	0.60**		
Terpene	0.08	0.10	-0.43*	0.05	-0.02	0.09	-0.04	
Thickness	-0.24	0.09	0.06	0.06	0.09	0.07	0.16	-0.19

Asterisks denote statistically significant correlations at *P* <0.05 (*), *P* < 0.01 (**), and *P* <0.001 (***).

The summary of flammability metrics provides an overview of fire behaviour within the burns (Table 5). Of the plots with attempted ignitions, 55% burnt and the average burn coverage within each burnt plot was 86%. The average char height across all the plots was 0.8 m and the average scorch height was 7 m.

**Table 5:** Summary of flammability metrics within each burn and overall. Standard deviations are shown in brackets after mean values.

	Aldermans Creek	Britannia Range	Mt Toolebewong	Overall
Plots with an ignition attempt	76	29	19	124
Plots burnt^1^	54	16	11	81
Mean scorch height (m)	9 (8)	4 (6)	5 (5)	7 (8)
Mean char height (m)	1.1 (0.7)	0.5 (0.7)	0.6 (0.6)	0.9 (0.7)
Mean percentage burnt (%)	90 (18)	69 (38)	28 (28)	85 (26)

^1^ These plots represent those which burnt *and* had present the combination of studied-species as the dominant flora

**Fig 4:**
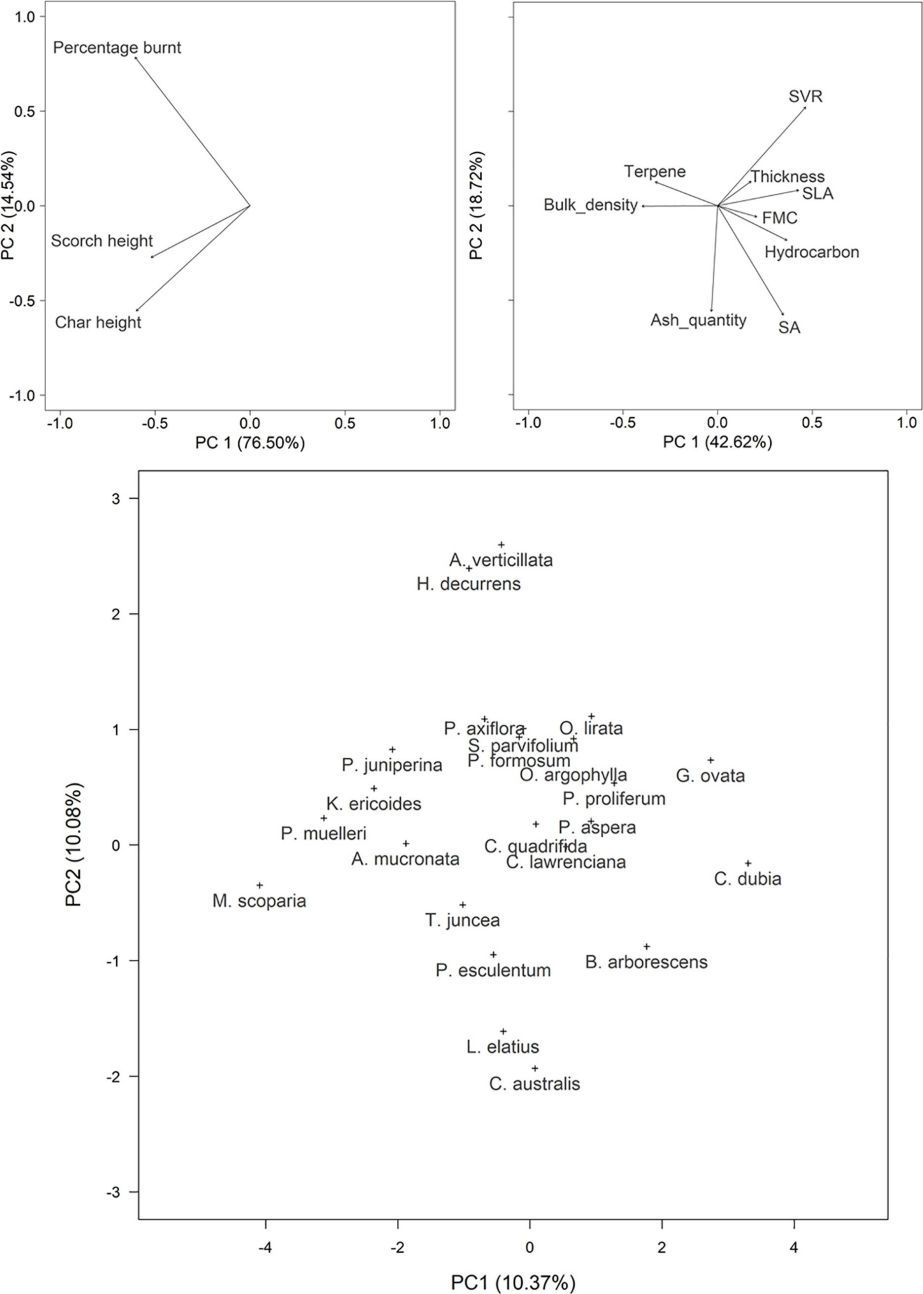
Constrained RLQ ordinations for (a) Flammability metrics–R, (b) Plant traits–Q and (c) Species composition–L. The ordination space is aligned for all ordinations, however the scale differs for the species composition ordination. SVR, surface area to volume ratio; SLA, specific leaf area; FMC, fuel moisture content; SA, surface area.

The RLQ analysis provides a visual summary of associations between plant traits at the field scale and their links with flammability metrics. The constrained RLQ output (Fig 4) is derived from the outputs of three independent ordinations for each data type; the individual ordinations explain 91%, 20% and 61% of the variability in the data for field flammability (R), species abundance (L) and trait data (Q), respectively (S1 Appendix). In the constrained RLQ, most of the variability is explained by the first axis (99%) (S2 Appendix). The positive values of the first RLQ axis indicates that species with higher SLA, hydrocarbon content and FMC, and reduced terpene content and bulk density (e.g. *C*. *dubia*, *G*. *ovata*, *P*. *proliferum*, *O*. *lirata*, *O*. *argophylla*, *C*. *lawrenciana* and *P*. *aspera* -Fig 4B and 4C) are associated with plots that had lower char and scorch heights and lower percentage of plot burnt (Fig 4A). Species associated with increased char and scorch heights and higher percentage of plot burnt (e.g. *M*. *scoparia*, *P*. *muelleri*, *K*. *ericoides*, *P*. *juniperina*, *A*. *mucronata*, *T*. *juncea*, *H*. *decurrens* and *P*. *axiflora–*Fig 4A and 4C) had higher terpene content and bulk density, and reduced SLA, hydrocarbon content and FMC (Fig 4B).

The fourth-corner method was used to test the bivariate associations between plant traits and flammability metrics. Out of the 27 possible associations, 9 were significant after correcting for repeated testing (*P* < 0.05, Table 6). Bulk density was positively correlated with the flammability metrics; as bulk density increased char height and percentage area burnt also increased. Hydrocarbons, SA, SLA and SVR were negatively correlated with one or more of the flammability metrics. As hydrocarbon content and SA increased, the percentage of the plot burnt decreased; as SLA increased, char height and the percentage of the plot burnt decreased; and as SVR increased, all flammability metrics decreased. There were no significant associations for the remaining plant traits. Overall, the global multivariate test was highly significant (*P* < 0.0001), indicating the presence of a relationship between species traits and field measures of flammability.

**Table 6:** Correlation coefficients as determined by the fourth-corner analysis. Significant (*P* < 0.05) positive associations are represented by orange cells and significant negative associations are represented by blue cells.

	Char height	Percentage burnt	Scorch height
Ash quantity	0.08	-0.01	0.01
Bulk density	0.36	0.34	0.26
Fuel moisture content	-0.19	-0.18	-0.12
Hydrocarbon	-0.30	-0.32	-0.26
Surface area	-0.25	-0.34	-0.25
Specific leaf area	-0.36	-0.35	-0.31
Surface area to volume ratio	-0.39	-0.34	-0.39
Terpene	0.26	0.29	0.24
Thickness	-0.16	-0.14	-0.12

Non-significant associations are blank. P-values were adjusted for multiple comparisons using FDR (false discovery rate) procedures.

## 4. Discussion

There is a substantial body of research linking plant traits to flammability metrics in the laboratory (as reviewed in [25] and [7]) and comparatively little research linking plant traits to flammability metrics in the field (one exception being [86]). Using a cross-scale approach, we found a significant association between field-scale flammability and some of the plant traits identified as important to flammability in the laboratory. These findings suggest that live vegetation traits, and accordingly species composition, may play a role defining how fires burn at field-scales and therefore warrant further consideration when contemplating fire behaviour across the landscape.

### 4.1 Species-level associations between plant traits

At the species level, there was a high degree of covariation in traits within individual plant species. From an evolutionary physiology perspective, this is unsurprising as multiple traits can confer fitness to the same selective pressures [87,88]. Interestingly, the laboratory-measured effects of these covarying traits were not consistently synergistic in terms of plant flammability. For example, FMC and SLA were positively correlated (r = 0.82, Table 4), however high FMC has been shown to reduce ignitability by increasing the energy required for ignition [10,29], whereas high leaf area (per unit of dry weight) has been shown to increase ignitability by increasing fuel heating efficiency [15,16]. Regarding the combined effect on multiple traits on the flammability of plants, we know little about the relative importance of each trait and how different traits interact to effect overall plant flammability; it is feasible that the contributions of a small number of traits could overwhelm the effects of other less influential traits. To resolve this further research is needed at the scale of entire plants to quantify the effect of multiple traits together.

### 4.2 Field-scale associations between plant traits

At the field level, species that co-occurred often exhibited similar traits, suggesting a plant community might have a common assemblage of traits. For example, species with positive values on the species ordination axis (e.g. *C*. *dubia*, *B*. *arborescens*, *P*. *proliferum* and *P*. *aspera*, Fig 4C) that are recognised as common components of Damp forests, were associated with greater SLA, FMC and hydrocarbons. Species that were negative on the first axis of the species ordination (e.g. *M*. *scoparia*, *P*. *muelleri*, *A*. *mucronata* and *K*. *ericoides*, Fig 4C) that are common in Heathy Dry Forest, had lower values in the same traits but higher terpene contents and bulk densities. Dickinson and Kirkpatrick [12] also reported higher FMC among wetter eucalypt forest species and lower FMC for drier forest species. As traits represent adaptations to selective pressures, it is not unexpected that species that co-occur would converge in trait combinations [87,88]. This convergence of plant flammability properties at the community level suggests that traits play a role in defining field-scale flammability and that species composition information could be used in the future to help predict fire behaviour. As the properties of plant communities can be predicted through space using biophysical models [89], this provides the potential for using such approaches to predict the contribution of the combined plant trait effect on fire behaviour, supplementing current approaches that consider fuel load and structure alone [8].

### 4.3 Linking plant traits to field-scale metrics of flammability

The fourth-corner analysis and RLQ showed a highly statistically significant relationship between plant traits and field-scale flammability. As few studies have evaluated the links between small-scale studies and field-scale outcomes (one exception being [90]), this key finding is one of the first to show that a relationship exists at this scale.

Five of the nine traits evaluated were significantly associated with flammability at field scales. Bulk density was positively associated with char height and the percentage of the plot burnt. Other studies have reported a parabolic relationship between bulk density and fire spread; too sparse and the fuel cannot propagate fire, too dense and it can restrict fuel aeration [24,91,92]. Our results capture only part of this parabolic curve, before the vegetation reaches the threshold density above which field-scale flammability is supressed. Surprisingly, SLA, SA and SVR, were negatively associated with the flammability metrics, despite the converse being expected from laboratory research conducted on individual live leaves. Although our result contradicts prior research, it is not surprising since increased SLA, SA and SVR represent species with large thin leaves, which are adaptations to low light [93], and lower light levels tend to occur beneath denser canopies in wetter or more sheltered parts of the landscape [64] where fires are known to be less intense [94]. High hydrocarbons were also associated with reduced field-scale flammability–these extractives are likely to be in the form of waxes on plant leaves which may be slower to ignite, as opposed to the more volatile terpene oils that have been focused on thus far. Hydrocarbons have not been a focus of prior laboratory flammability research, but our results suggest they warrant further consideration.

Also noteworthy were the plant traits that were not found to have strong associations with flammability at field scales. There was no association between the flammability metrics and terpenes, despite many studies alluding to the importance of these volatile compounds and laboratory studies showing that increasing yields of terpenes can increase ignition potential (e.g. [14,35]). This result may reflect the relatively low terpene contents we measured among the understorey species in this study (S2 Table) as compared to the higher ranges found in prior studies; much higher terpene contents may be required before they have a significant effect on field-scale flammability. The FMC of live plants was not found to be associated with any of the flammability metrics, which is another surprising result as other studies identify it as a key trait influencing plant flammability in the laboratory [17,37,40]. The lack of an effect in our study could reflect differences in heat flux between the field and laboratory as the influence of live FMC on field-scale flammability has been shown to decrease as the heat source increases [53,95,96]. Alternatively, the moisture values measured may not have been representative of conditions during the prescribed burns, since the plants were sampled for trait analysis at a different time to the burns or the live fuel moisture effect may have been overwhelmed by the effects of other traits.

Although strong links between some plant traits and field-scale flammability were demonstrated, it is important to highlight that these links represent associations, not causal links. The flammability metrics (percentage area burnt, char and scorch height) measured in the field depend on the flammability of the entire system, which is likely to include plant traits and other factors including the amount and properties of dead fuels (e.g. surface litter, bark and suspended dead fuels [97]), exposure to solar radiation and air movement (which can be influenced by topography and the overstory canopy; [63,64,98] and dead fuel moisture (which can be a function of humidity, landscape position and vegetation structure; [99–101]. If more data, such as a wider range of field-scale flammability measurement (e.g. rate of spread), were collected across more prescribed burns and a wider range of weather conditions, then there would be more potential to isolate the contribution and importance of particular plant traits to fire behaviour. That said, our analysis demonstrates an approach that can be used to bridge the gap between the laboratory and field, to start building a clearer understanding of which plant traits influence field-scale flammability. Many different environmental pressures can lead to changes in the composition of species, e.g. invasive species, disturbance history and climate change. Understanding how this may drive changes to the flammability of landscapes will be important to understanding the likely nature of future fire regimes and allow managers to better target interventions to manage wildfire risk.

## Conclusion

Many studies consider links between plant traits and flammability in the laboratory while comparatively few consider these relationships at field scales. In this study we sought to bridge the gap between the laboratory and landscape by linking plant traits to metrics of field-scale flammability. We found a high number of traits exhibited co-variation at the species-level and that species with similar trait profiles occurred together in the field. There was a significant relationship between some plant traits (bulk density, hydrocarbons, specific leaf area and surface area to volume ratio) and field-scale metrics of flammability, suggesting that plant traits are associated with flammability at field scales. This result highlights a need for further research to better understand the role of vegetation community composition in driving fire behaviour. Our study successfully demonstrates a method that can be used to start bridging the gap between the laboratory and the field.

## Supporting information

S1 Table Summary of daily weather, from the Coldstream weather station, during the prescribed burns. (DOCX)

S2 Table Summary of measured plant traits for each species. (DOCX)

S1 Appendix Summary of outputs for individual ordinations. (DOCX)

S2 Appendix Summary of outputs for the constrained RLQ ordination. (DOCX)

S1 Dataset Field data from the prescribed burns. (XLSX)
