# Correction: Plant traits linked to field-scale flammability metrics in prescribed burns in *Eucalyptus* forest

**DOI:** 10.1371/journal.pone.0223401

**Published:** 2019-09-30

**Authors:** The PLOS ONE Staff

The second sentence of the caption for Table 1 incorrectly appears as a subsection of the Introduction titled “The direction of correlation with the plant trait and the flammability metric tested is shown in brackets.” Please see the complete, correct Table 1 caption here.

**Table 1:** Summary of plant flammability findings for laboratory studies of live plant traits. The direction of correlation with the plant trait and the flammability metric tested is shown in brackets.

Plant trait	Definition of trait	Correlation with plant flammability	Works describing the plant trait and plant flammability association
Ash quantity (%)	Percent of mineral content remaining after combustion determined as a ratio of oven dry weight	(-) combustibility	[11,12,27–30]
Bulk density^1^ (g cm^3^)	Amount of oven dry biomass per volume of airspace	Undetermined in the laboratory	[24,25,31,32]
Extractives^2^ (mg gDW^1^)	Amount of volatile compounds including waxes, fats, oils and terpenes. Typically, only terpenes are considered, or no distinction is made between terpenes and other volatile compounds.	(+) ignitability (+) combustibility	[12,14,33–37]
Fuel moisture content^3^ (%)	Percent of water in a plant organ determined as a ratio of dry weight.	(-) ignitability (-) combustibility	[16,17,30,37–41]
Specific leaf area (cm^2^ g^-1^)	The one-sided area of a fresh leaf, divided by its oven dry weight	(+) ignitability	[15,16,42]
Surface area (cm^2^)	The total area of a plant organ, typically leaves	(+) ignitability	[16,43–45]
Surface area to volume ratio (cm^-1^)	Ratio between surface area and total volume of a plant organ	(+) ignitability (+) combustibility	[17,29,46,47]
Thickness (cm)	Width of the plant organ	(-) ignitability	[13,48]

^1^ Bulk density has a parabolic relationship with flame spread rate; it can be aeration or fuel limiting and its effect depends on the fuel layer being investigated. It has been extensively studied in the laboratory for leaf litter.

^2^ Several plant flammability studies that include volatile compounds are inconclusive about their importance.

^3^ The effects of fuel moisture content (FMC) is not always consistent, as some live fuels can burn intensely at high FMC.

The third and fourth sentences of the caption for Table 6 incorrectly appear below the table. Please see the complete, correct Table 6 caption here.

**Table 6:** Correlation coefficients as determined by the fourth-corner analysis. Significant (*P* < 0.05) positive associations are represented by orange cells and significant negative associations are represented by blue cells. Non-significant associations are blank. P-values were adjusted for multiple comparisons using FDR (false discovery rate) procedures.

	Char height	Percentage burnt	Scorch height
Ash quantity	0.08	-0.01	0.01
Bulk density	0.36	0.34	0.26
Fuel moisture content	-0.19	-0.18	-0.12
Hydrocarbon	-0.30	-0.32	-0.26
Surface area	-0.25	-0.34	-0.25
Specific leaf area	-0.36	-0.35	-0.31
Surface area to volume ratio	-0.39	-0.34	-0.39
Terpene	0.26	0.29	0.24
Thickness	-0.16	-0.14	-0.12

The publisher apologizes for these errors.
