# Supplementary material for: Plant traits linked to field-scale flammability metrics in prescribed burns in Eucalyptus forest
Source: PLoS One. 2019 Aug 26;14(8):e0221403. doi: 10.1371/journal.pone.0221403 (PMC6709903; doi:10.1371/journal.pone.0221403)
Supplement: S1 Table — (DOCX) [file pone.0221403.s001.docx]

|  | Burn dates | Max temperature (°C) | | Min RH | | Max FFDI | | Max KBDI | | Max wind speed (km/h) | |
| --- | --- | --- | --- | --- | --- | --- | --- | --- | --- | --- | --- |
| Toolebewong | 19-Apr-17 | 26.3 | 41 | | 11 | | 100.7 | | 11 | |  |
|  | 20-Apr-17 | 25.8 | 38 | | 13 | | 101.9 | | 12 | |  |
| Brittania | 18-Apr-16 | 25 | 39 | | 11 | | 138.6 | | 11 | |  |
|  | 19-Apr-16 | 26.5 | 32 | | 15 | | 139.6 | | 11 | |  |
| Aldermans | 29-Mar-16 | 19.1 | 55 | | 6 | | 129 | | 26 | |  |
|  | 4-Apr-16 | 24.7 | 38 | | 8 | | 132 | | 11 | |  |
|  | 14-Apr-16 | 26.4 | 31 | | 16 | | 136.6 | | 12 | |  |
|  | 15-Apr-16 | 27.1 | 23 | | 22 | | 137.4 | | 12 | |  |
|  | 18-Apr-16 | 25 | 39 | | 11 | | 138.6 | | 11 | |  |
|  | 19-Apr-16 | 26.5 | 32 | | 15 | | 139.6 | | 11 | |  |
| Averages |  | 25.24 | | 368 | | 12.8 | | 129.4 | | 12.8 | |

# S1 Table: Summary of daily weather, from the Coldstream weather station*, during the prescribed burns. RH, relative humidity; FFDI, forest fire danger index; KBDI, Keetch-Byram Drought Index

*The Bureau of Meteorology details for the Coldstream weather station; Station ID number 086383, Lat: -37.72, Lon: 145.41, Height” 83.0 m
