# Supplementary material for: Plant traits linked to field-scale flammability metrics in prescribed burns in Eucalyptus forest
Source: PLoS One. 2019 Aug 26;14(8):e0221403. doi: 10.1371/journal.pone.0221403 (PMC6709903; doi:10.1371/journal.pone.0221403)
Supplement: S2 Table — (DOCX) [file pone.0221403.s002.docx]

# S2 Table: Summary of measured plant traits for each species^1^

|  | Ash quantity (% ash) | Bulk density (g cm^3^) | FMC (% odw) | Hydro-carbon (mL g^-1^) | Surface area (cm^2^) | Specific leaf area (cm^2^ g^-1^) | Surface area to volume ratio | Terpene (mg gDW^-1^) | Thickness (cm) |
| --- | --- | --- | --- | --- | --- | --- | --- | --- | --- |
| Acacia mucronata | 0.10 (0.02) | 0.38 (0.11) | 125.46 (3.07) | 1.34 (0.49) | 25.01 (3.94) | 51.13 (13.09) | 30.37 (7.78) | 0.82 (0.26) | 0.034 (0.00) |
| Acacia verticillata | 0.09 (0.01) | 0.24 (0.13) | 131.22 (24.12) | 3.13 (1.66) | 0.06 (0.01) | 115.34 (20.49) | 76.73 (30.67) | 0.26 (0.15) | 0.04 (0.00) |
| Bedfordia arborescens | 0.12 (0.00) | 0.20 (0.06) | 377.32 (46.32) | 9.58 (1.60) | 78.54 (16.79) | 394.15 (39.72) | 27.81 (0.84) | 1.37 (0.48) | 0.04 (0.00) |
| Calochlaena dubia | 0.10 (0.01) | 0.10 (0.02) | 190.78 (4.76) | 11.48 (1.00) | 642.67 (186.82) | 237.21 (12.57) | 67.32 (2.08) | 0.00 (0.00) | 0.06 (0.09) |
| Coprosma quadrifida | 0.12 (0.02) | 0.12 (0.04) | 139.04 (5.41) | 3.00 (0.74) | 0.46 (0.12) | 127.19 (40.74) | 46.31 (16.68) | 0.00 (0.00) | 0.01 (0.00) |
| Correa lawrenciana | 0.14 (0.01) | 0.14 (0.02) | 167.28 (21.54) | 2.8 (1.25) | 23.44 (7.31) | 242.17 (32.12) | 60.93 (5.54) | 1.80 (0.65) | 0.04 (0.00) |
| Cyathea australis | 0.17 (0.01) | 0.31 (0.08) | 188.61 (21.81) | 3.17 (1.01) | 1346.86 (501.78) | 199.69 (55.69) | 51.15 (10.41) | 0.23 (0.16) | 0.02 (0) |
| Goodenia ovata | 0.17 (0.02) | 0.1 (0.05) | 260.51 (24.91) | 1.54 (0.07) | 45.03 (12.44) | 373.16 (20.75) | 114.28 (17.84) | 0.75 (0.14) | 0.04 (0.01) |
| Hakea decurrens | 0.07 (0.01) | 0.43 (0.12) | 123.03 (7.16) | 0.2 (0.19) | 0.51 (0.09) | 99.26 (5.4) | 47.65 (10.85) | 0.02 (0.04) | 0.11 (0.02) |
| Kunzea ericoides | 0.12 (0.01) | 0.25 (0.11) | 109.09 (2.84) | 1.95 (0.13) | 0.21 (0.03) | 55.07 (22.53) | 29.72 (11.97) | 7.99 (1.22) | 0.01 (0) |
| Lepidosperma elatius | 0.15 (0) | 0.13 (0.06) | 138.38 (28.38) | 1.96 (0.46) | 326.68 (91.52) | 101.27 (28.4) | 30.69 (5.89) | 0.28 (0.1) | 0.04 (0.01) |
| Monotoca scoparia | 0.15 (0.01) | 0.81 (0.15) | 77.76 (3.23) | 1.46 (1.2) | 0.18 (0.03) | 37.93 (4.07) | 27.13 (8.75) | 0.3 (0.12) | 0.03 (0) |
| Olearia argophylla | 0.09 (0) | 0.2 (0.07) | 153.84 (29.03) | 3.67 (0.77) | 95.46 (31.58) | 209.19 (23.23) | 66.91 (4.92) | 0.54 (0.13) | 0.03 (0) |
| Olearia lirata | 0.09 (0) | 0.1 (0.05) | 193.82 (18.01) | 3.01 (0.32) | 26.63 (4.75) | 322.99 (61.53) | 56.69 (1.74) | 6.27 (1.43) | 0.03 (0) |
| Pimelea axiflora | 0.09 (0.01) | 0.12 (0.05) | 127.39 (46.93) | 1.56 (0.24) | 2.96 (1.31) | 119.16 (11.84) | 47.18 (1.51) | 0.4 (0.16) | 0.02 (0) |
| Platylobium formosum | 0.11 (0.01) | 0.14 (0.05) | 113.22 (8.83) | 0.37 (0.13) | 12.46 (6.98) | 160.01 (23.23) | 65.41 (6.88) | 1.28 (0.26) | 0.03 (0.01) |
| Polystichum proliferum | 0.14 (0.01) | 0.15 (0.07) | 192.06 (18.75) | 2.97 (1.82) | 33.67 (6.54) | 262.55 (34.52) | 86.82 (9.58) | 0.76 (0.25) | 0.02 (0) |
| Pomaderris aspera | 0.14 (0) | 0.17 (0.05) | 161.48 (27.07) | 0.93 (0.31) | 63.71 (15.27) | 270.86 (52.6) | 62.78 (8.5) | 0.3 (0.3) | 0.06 (0.01) |
| Pteridium esculentum | 0.14 (0) | 0.24 (0.07) | 146.81 (4.51) | 1.2 (0.13) | 184.94 (41.05) | 115.12 (7.01) | 41.65 (1.67) | 0.28 (0.08) | 0.04 (0) |
| Pultenaea juniperina | 0.1 (0.02) | 0.29 (0.14) | 150.08 (27.62) | 0.39 (0.15) | 0.33 (0.06) | 51.51 (21.83) | 20.55 (5.67) | 0.29 (0.11) | 0.04 (0.01) |
| Pultenaea muelleri | 0.12 (0.01) | 0.45 (0.11) | 86.41 (9.26) | 0.03 (0.06) | 0.15 (0.04) | 66.87 (9.8) | 25.41 (3.11) | 0.18 (0.13) | 0.01 (0) |
| Spyridium parvifolium | 0.09 (0.01) | 0.07 (0.02) | 146.27 (16.14) | 1.53 (0.3) | 4.49 (1.53) | 213.21 (40.08) | 48.94 (22.03) | 1.19 (0.2) | 0.02 (0) |
| Tetrarrhena juncea | 0.13 (0) | 0.25 (0.14) | 196.26 (51.71) | 1.58 (0.29) | 6.9 (1.46) | 130.47 (24.53) | 34.44 (5.64) | 0.12 (0.11) | 0.01 (0) |

# ^1^ Values are means with 1 standard error in parentheses
