# Supplementary material for: Plant traits linked to field-scale flammability metrics in prescribed burns in Eucalyptus forest
Source: PLoS One. 2019 Aug 26;14(8):e0221403. doi: 10.1371/journal.pone.0221403 (PMC6709903; doi:10.1371/journal.pone.0221403)
Supplement: S1 Appendix — (DOCX) [file pone.0221403.s003.docx]

# S1 Appendix: Summary of outputs for individual ordinations

Burn measurement across sites (R)

Total inertia: 3

|  | **Ax1** | **Ax2** | **Ax3** |
| --- | --- | --- | --- |
| **Eigenvalues** | 2.30 | 0.44 | 0.27 |
| **Projected inertia (%)** | 76.50 | 14.54 | 8.96 |

|  | **Ax1** | **Ax1:2** | **Ax1:3** |
| --- | --- | --- | --- |
| **Cumulative projected inertia (%):** | 76.50 | **91.04** | 100.00 |

Species abundance across sites (L)

Total inertia: 7.5

|  | **Ax1** | **Ax2** | **Ax3** |
| --- | --- | --- | --- |
| **Eigenvalues** | 0.78 | 0.76 | 0.70 |
| **Projected inertia (%)** | 10.37 | 10.08 | 9.32 |

|  | **Ax1** | **Ax1:2** | **Ax1:3** |
| --- | --- | --- | --- |
| **Cumulative projected inertia (%):** | 10.37 | **20.44** | 29.76 |

Traits across species (Q)

Total inertia: 9

|  | **Ax1** | **Ax2** | **Ax3** |
| --- | --- | --- | --- |
| **Eigenvalues** | 3.84 | 1.68 | 1.10 |
| **Projected inertia (%)** | 42.62 | 18.72 | 12.17 |

|  | **Ax1** | **Ax1:2** | **Ax1:3** |
| --- | --- | --- | --- |
| **Cumulative projected inertia (%):** | 42.62 | **61.34** | 73.51 |
