# Supplementary material for: Plant traits linked to field-scale flammability metrics in prescribed burns in Eucalyptus forest
Source: PLoS One. 2019 Aug 26;14(8):e0221403. doi: 10.1371/journal.pone.0221403 (PMC6709903; doi:10.1371/journal.pone.0221403)
Supplement: S2 Appendix — (DOCX) [file pone.0221403.s004.docx]

# S2 Appendix: Summary of outputs for the constrained RLQ ordination

Total inertia: 1.94

|  | **Ax1** | **Ax2** | **Ax3** |
| --- | --- | --- | --- |
| **Eigenvalues** | 1.92 | 0.01 | 0.01 |
| **Projected inertia (%)** | **99.01** | 0.55 | 0.34 |

|  | **Ax1** | **Ax1:2** | **Ax1:3** |
| --- | --- | --- | --- |
| **Cumulative projected inertia (%):** | 99.09 | **99.64** | 100.00 |

Eigenvalues decomposition:

|  | **eig** | **covar** | **sdR** | **sdQ** | **corr** |
| --- | --- | --- | --- | --- | --- |
| **1** | 1.92 | 1.39 | 1.51 | 1.91 | 0.48 |
| **2** | 0.01 | 0.10 | 0.65 | 1.05 | 0.15 |

Inertia & coinertia R (burn):

|  | **inertia** | **max** | **ratio** |
| --- | --- | --- | --- |
| **1** | 2.28 | 2.30 | 0.99 |
| **1+2** | 2.70 | 2.73 | **0.991** |

Inertia & coinertia Q (traits):

|  | **inertia** | **max** | **ratio** |
| --- | --- | --- | --- |
| **1** | 3.64 | 3.84 | 0.95 |
| **1+2** | 4.74 | 5.52 | **0.858** |

Correlation L (species):

|  | **corr** | **max** | **ratio** |
| --- | --- | --- | --- |
| **1** | **0.48** | 0.88 | 0.55 |
| **2** | 0.15 | 0.87 | **0.174** |
